# ‘*Knowing as we go*’: a Hunter-Gatherer Behavioural Model to Guide Innovation in Sport Science

**DOI:** 10.1186/s40798-020-00281-8

**Published:** 2020-10-28

**Authors:** Carl T. Woods, Sam Robertson, James Rudd, Duarte Araújo, Keith Davids

**Affiliations:** 1 Institute for Health and Sport, Victoria University, Melbourne, Australia; 2 Research Institute for Sport and Exercise Sciences, Liverpool John Moores University, Liverpool, UK; 3 CIPER, Faculdade de Motricidade Humana, Universidade de Lisboa, Lisbon, Portugal; 4 Sport & Human Performance Research Group, Sheffield Hallam University, Sheffield, UK

**Keywords:** Ecological dynamics, Social anthropology, Transdisciplinarity, Knowledge of/about, Self-regulation, Sports performance

## Abstract

Where do novel and innovative ideas in sport science come from? How do researchers and practitioners collectively explore the dynamic landscape of inquiry, problem, solution and application? How do they learn to skilfully navigate from current place and practice toward the next idea located beyond their current vantage point? These questions are not just of philosophical value but are important for understanding how to provide high-quality support for athletes and sport participants at all levels of expertise and performance. Grounded in concepts from social anthropology, and theoretically positioned within an ecological dynamics framework, this opinion piece introduces a hunter-gatherer model of human behaviour based on wayfinding, situating it as a conceptual guide for implementing innovations in sport science. Here, we contend that the embedded knowledge of a landscape that guides a successful hunting and gathering party is germane to the pragmatic abduction needed to promote innovation in sport performance, leading to the inquisition of new questions and ways of resolving performance-preparation challenges. More specifically, exemplified through its transdisciplinarity, we propose that to *hunt* ‘new ideas’ and *gather* translatable knowledge, sport science researchers and practitioners need to wayfind through uncharted regions located in new performance landscapes. It is through this process of navigation where individuals will deepen, enrich and grow current knowledge, ‘taking home’ new ideas as they find their way.

## Key Points

- Grounded in social anthropology, and theoretically positioned within ecological dynamics, this opinion piece introduces a hunter-gatherer model of human behaviour based on wayfinding, situating it as a conceptual guide for innovation in sport science.
- The embedded knowledge of a landscape that guides a hunting and gathering party is germane to the pragmatism needed to promote innovation in the science underlying sport performance.
- A threaded hypothetical example of the proposed hunter-gatherer model is offered, anchored around three anthropological principles.

## Introduction

*“…as for me, I am tormented with an everlasting itch for things remote. I love to sail forbidden seas, and land on barbarous coasts”*—Herman Melville in Moby Dick.

The desire to explore uncharted regions within a landscape is a common thread that links humans across different epochs and cultures. In life, it is this sense of exploration that leads us to travel, move to a new city, or venture off the trail during a bushwalk. Similarly, in science, it is this sense of exploration that drives the establishment of new perspectives—manifesting—for example, in the emergence of innovative ideas seen in landscape architecture [1], computational software [2], engineering [3], and education [4]. In each of these examples, researchers have sought to grow ‘disciplinary’ knowledge through the development of frameworks that promote abductive and transdisciplinary thinking, which ultimately functions to instil innovative *ways of doing*.

Indeed, despite still being in its relative infancy, sport science is no different to these disciplines. Innovation is praised and sought after, exemplified through the establishment of Research and Development departments in high-level sports organisations, and seen through the offering of unique, international, educational forums. It is also explicitly stated within the scope of some of sport science’s highest-ranked scientific journals. However, while there appears to be a *want* for innovation in sport science, there is a lack of support to guide such processes, perhaps leaving researchers and practitioners bereft of knowing how to innovate or where to start. This limitation, in part, may explain why sport science has largely been confined to insular thinking and interaction [5–8]. Within research, this has been typified through reductionistic approaches to solving problems isolated within ‘sub-disciplines’ such as biomechanics and physiology [6–10]. While within practice, although there may be an extensive range of specialist, discipline-based sections structured into sports organisations to support athlete development and performance (e.g. strength and conditioning, high-performance, performance analysis, and coaching departments), their functioning is often driven by isolated, silo-based thinking, limiting their capacity to integrate activities to fulfil organisational objectives [11, 12].

Moreover, there is a tendency in scientific inquiry to follow previously defined procedures, where exploratory behaviour is denied. Reed [13] went as far as saying that scientific thought has largely abandoned firsthand human experience and that, consequently, wayfinding (i.e., the process of learning to navigate through unfamiliar ‘regions’ in a landscape of knowledge and experiences by connecting to the environment), contrasting with mechanistic Cartesian views, has become devalued on all fronts. These predominant mechanistic views foster a more reflective, mediating intellectualism, amplified by the distancing and de-contextualising effects of a great deal of modern technology (i.e., when decisions of how to proceed are indicated by software algorithms, if a ‘system is not down’). Thus, the tendency is to become a nexus of heavily processed experience, where science is produced and sold in pre-interpreted, packaged, and anonymous forms that degrade our abilities to directly encounter and experience information [13]. It is perhaps why Balagué et al. [8] encouraged academic sport scientists to abandon the short-term fixation on things that could lead to insularity, such as generating publications and/or obtaining academic promotions, in replace for a more authentic integration of mystery, exploration and an embracement of the unknown.

In light of this, both in research and practice, work has called for sport to progress beyond such reductionism and embrace interdisciplinarity [6, 14–16], progress toward transdisciplinarity [5, 8, 11], and even establish a unified way of thinking [10]. For example, Glazier [10] argued for a Grand Unified Theory of sports performance based on the constraints framework introduced by Newell [17]. Further, Rothwell et al. [11] conceptualised how a Department of Methodology (DoM) could underpin applied sport science to avoid ‘system capture’ and promote greater transdisciplinarity in practice by removing siloed and insular thinking (also see [12]). Accordingly, sport science appears to be on the cusp of a new way of thinking, one that promotes ‘upward, outward and collaborative’ inquisition, encouraging the search for *ways* to navigate beyond the path dependant and traditional confines that have led to this point [7–11].

In an attempt to support this emerging consciousness and drive new ideas, grow knowledge and promote pragmatic transdisciplinarity in sport, this opinion piece introduces a conceptual model epistemologically situated within social and biophysical anthropology [18] and ecological psychology [13, 19]. Specifically, inspired by Steinert and Leifer [3], this opinion piece introduces a hunter-gatherer model based on wayfinding as a conceptual basis to guide innovation in sport science. Distinctions between hunting and gathering instincts are made, demonstrating the importance of both when navigating through uncharted regions of the performance landscape in the pursuit of *new ways of doing*. This paper encourages sport scientists to re-discover their hunter-gatherer instincts, leaving behind the normative or pre-processed ways of navigating knowledge (based on fixed, sequentially pre-planned and compartmentalised approaches that are structured to lead to ‘known’ destinations). Instead, they are urged to set out along an emergent path of innovation, navigating through new and uncharted regions, embedding, enriching and growing knowledge as they find their way.

## Innovation and Knowledge ‘Growth’

### Exploring a Complex and Uncertain Landscape

In this paper, we do not wish to situate innovation as the process of simply thinking differently about disciplinary problems, nor do we contend that innovation is solely localised to the realm of technologies or product development. Instead, it is proposed that innovation should be situated more generally, incapsulating both the development of new technologies and methodologies, and the formulation of new ideas that may diverge to a positive progression from traditional ways of doing, thinking and problem-solving. In both respects, innovation should not be viewed as a linear or predictable process that one can chart a course to a terminus destination. Rather, innovation is an evolving, complex, uncertain and disorderly process, which emerges and changes, based on a range of dynamically interacting constraints [20, 21]. More specifically, it can be understood as a set of abductive movements that emerge within a complex adaptive system, functioning in degenerative ways to navigate toward new ways of doing that evolve along timescales of performance, learning and development [22]. Simply, innovation is not something to be ‘acquired’ or that originates in the isolated brain. Instead, innovation emerges in the practical engagement with materials and people [21, 23]. It is a continually adaptive, transdisciplinary and evolving process that unfolds by (in)directly digressing from engrained and culturally pervasive disciplinary norms [21, 22].

To support this view of innovation, more contemporary conceptual models tend to be underpinned by a chain-linked approach [21]. This approach contends that there is no one (linear) path toward innovation, but numerous potential trajectories that abduct in all directions, unfolding as individuals immerse themselves in new information [20–23]. The interaction with this new information is what leads to the *growth* of knowledge, affording individuals opportunities to (re)organise action in real-time as they move along an evolving path. This contention draws support from Gibson’s [24] differentiation of knowledge—defined as *knowledge of* and *knowledge about* the environment. To know *about* an environment is to have general knowledge about a state of affair, manifested in verbalised responses to questions or in the presentations of pictures or symbols. Comparatively, *knowledge of* one’s environment relates to an enmeshment between an individual, an environment and its many socio-materialistic things (i.e., norms, rules), exemplified through skilful perception and action that enables the achievement of a goal-directed outcome [25]. It is the latter of these knowledge types that requires an embedded and embodied understanding of a performance environment, and its possibilities for action, in sport [24].

Some key principles of the chain-linked approach should be emphasised with respect to innovation. First, it embraces the *uncertainty* of innovation. This directly implicates one’s (in)ability to pre-plan a route from ‘point A’ (i.e., current knowledge) to ‘point B’ (i.e., innovative idea) [21]. To innovate, one must, therefore, be willing to submit to a social anthropological ethos of *knowing as we go* [18]. This process of learning will inevitably take time, requiring one to *inhabit* [26, 27] new regions. However, through this inhabitation process, one will likely grow and embed his/her knowledge [26, 27], which helps prevent him/her from reaching *false summits*^1^ [21]. This does not mean that planning should be avoided when setting out to innovate. Contrarily, innovation requires pragmatic abduction guided by continually grown and embedded transdisciplinary knowledge. Ideas and planning can act as constraints, not instructions, in the relationship between researcher and materials [22, 23]. Further, the *cooperative* undertones of the chain-linked approach indicate that to embark upon a journey of innovation, collaborative, collective and complementary teamwork is required [20, 21]. This collective ethos was captured by Steinert and Leifer [3], who situated innovation as being an evolving process of *hunting* new ideas and *gathering* ways of implementing them. It is this cyclical process, resulting in the reconciliation of previously independent theories, methodologies, procedures and/or measurement tools, which ultimately leads toward the resolution of new, transdisciplinary ways of doing [2, 3].

In short, innovation should be viewed as a collective, evolutionary, interactive and emergent process that is often unpredictable, chaotic, disorderly and continuously subject to constraints—it is not the implementation of an idea, or an instructional recipe for the action of the researcher/practitioner. Conceptual models that are positioned to guide it should subsequently: (i) account for this unpredictability and uncertainty by avoiding a cartographic map that linearly navigates toward an ‘acquirable’ idea (terminus destination), (ii) encourage active exploration to generate and detect new transdisciplinary information to grow knowledge along the way, and (iii) promote collaboration and cooperation to enmesh previously independent theories and methodologies. Accordingly, in order for such a model to be of use to instil innovation in sport science, it must first sit within a theoretical framework that enables ongoing commitment to these principles.

## Ecological Dynamics

### A Transdisciplinary Framework to Instil Innovative Ways of Doing in Sport

Differing from interdisciplinarity and multidisciplinarity, transdisciplinarity calls for a “space of knowledge beyond the disciplines” [28, p.2]. Within such a ‘space’, previously independent theoretical and methodological constructs can be blended to afford inquisitive and collaborative thinking, allowing researchers and practitioners in sport the opportunity to ask new questions, and work toward the solving of existing problems viewed through a different lens [29, 30]. Moreover, transdisciplinarity extends beyond abstractions of knowledge, calling for integration, interaction and engagement between the inquirer and inquiry [28]. These ideas are important for understanding the broader contentions of this paper, with Balagué et al. [8] and Hristovski et al. [30] suggesting that the future of sport science is not within continued (sub-disciplinary) specialisations, but in the integration of seemingly disparate disciplines and paradigms, functioning to enrich (sport) scientific inquiry through the emergence of new, blended, and ultimately transdisciplinary, ways of doing. Importantly, however, this integration should be bound, or ‘glued’, by an overarching theoretical or conceptual framework that enables disparate disciplines to function collaboratively, thereby enriching one another while working toward a common goal.

Ecological dynamics offers sport such a transdisciplinary framework through its integration of concepts from ecological psychology [13, 19], constraints on dynamical systems [17, 31], the complexity sciences [32], evolutionary science [33], and social anthropology [34]. Within this framework, related concepts, such as skilled behaviour, learning, expertise and talent, are viewed as emergent properties of functionally adaptable and evolving performance solutions formed through the reciprocal relationship between a performer and the constraints of his/her environment [33]. Specifically, skilled actions are viewed as embedded, encultured, dynamic, body-environment interactions, which are self-organised by an individual as (s)he attempts to achieve a task goal.

In ecological dynamics, behavioural self-organisation refers to the development and exploitation of intertwined relationships that emerge between an individual’s actions, perceptions, cognitions, emotions and a performance environment [33, 35]. It is this particular aspect that has the potential to enrich a conceptual model used to guide innovation in sport. Specifically, the learning process in this framework has recently been conceptualised as wayfinding [36], a social anthropological concept describing an embedded process by which individuals learn to skilfully navigate through uncharted ‘regions’ in a landscape supported by a finely tuned perception-action coupling [18, 19, 27]. As discussed in greater depth below, there is no pre-determined route in which a learner follows when wayfinding; rather, they embark upon a self-regulating journey, progressively deepening and growing his/her *knowledge of* a landscape as they go, framed by the intentionality to traverse from one ‘region’ of the surroundings to another. This progressive entanglement between a wayfinder and his/her environment is not isolated to interactions with its physical features, but also relates to history, norms, social happenings and cultural rules, each enmeshing (with physical environmental features) to shape the way the learner navigates (self-regulates) through a performance landscape [37]. These perspectives are what we contend could lead to the requisite inhabitation and transdisciplinarity that ultimately instils innovative ways of doing.

## Wayfinding

Wayfinding is an ecological-anthropological concept that should be understood as a means of intentionally navigating along an evolving ‘path’, negotiating and reorganising passages *as* one goes, not *before* one goes [18]. In sports innovation, these ideas can be captured by an organisation’s intentionality, which frames the wayfinding activity undertaken. This perspective could guide the search of a sports organisation toward information-rich regions that afford opportunities for knowledge growth, ultimately leading toward the instilment of innovative ways of doing. The search for functionally innovative performance solutions is, therefore, not a completely random process, but is framed by the overarching intentions and how they relate to the innovation sought. For example, in the team sport of soccer, although an explicit plan to innovate may not be devised (which would reflect a more cartographic, linear approach), the overarching intention of promoting player exploration, creativity and adaptability may be sought, manifesting in innovative ways of redesigning practice tasks. How this innovative *way of doing* is achieved, though, requires wayfinding skills to seek and exploit available affordances (opportunities for action [19]) to redesign training to better support performance.

It is important to acknowledge that this innovative-prone intention may very well challenge historical and sociocultural norms, which would require careful consideration by those challenging them to ensure that genuinely good practices are maintained/instilled. Such constraints on innovation emphasise the importance of unbounded contemporality, with organisations needing to actively promote creative thought, inquisition and exploration through carefully regulated social norms that are not compelled by traditional or historical ways of doing [38]. While explicit strategies on ways to navigate away from encountered path-dependent behaviours are outside the scope of this opinion piece (interested readers should consult [39]), an example may be seen in the establishment of Research and Development departments within high-level sports organisations that function to normalise and actively encourage innovation, grounding practice within a contemporary theoretical framework for performance and learning, like ecological dynamics (see case example 2 in [40]). Thus, in an ecological conceptualisation of innovation in sport as a process of search, discovery and exploitation, skilful wayfinders could be individuals who are constantly responsive to the opportunities for action offered by the performance environment, learning to exploit these opportunities framed by the innovation-prone intention, and are supported by the sociocultural norms embedded into an organisation’s *form of life* [39].

### Hunter-Gatherers and Knowing as We Go

Bird-David [41] describes hunter-gatherers as individuals who view the world as an ‘integrated entity’, embedding themselves within their landscapes. It is this embedded, inhabitant knowledge, developed through rich interactions and engagement, which enables hunter-gatherers to grow their understanding, exploiting it intentionally to wayfind through vast distances between regions without the use of advanced technological aids [18]. Linking this description back to the aims of this opinion piece, hunter-gatherers wayfind through uncharted regions of a landscape not by following a pre-determined route on a map, but by exploiting the knowledge grown and experiences gained along the way. In this respect, a hunter-gatherer behavioural model based on wayfinding would not offer a map that leads to an innovative idea for researchers and practitioners, but would offer a transdisciplinary epistemology that promotes constant and evolving principles for ‘growth of knowledge’. Indeed, the core principles of such a model would advocate *search*, *intentionality*, *movement*, *observation*, *attunement*, *reflection* and *real-time (re)organisation of action* [3].

## A Hunter-Gatherer Model Based on Wayfinding to Guide Innovation in Sport Science

**Fig. 1:**
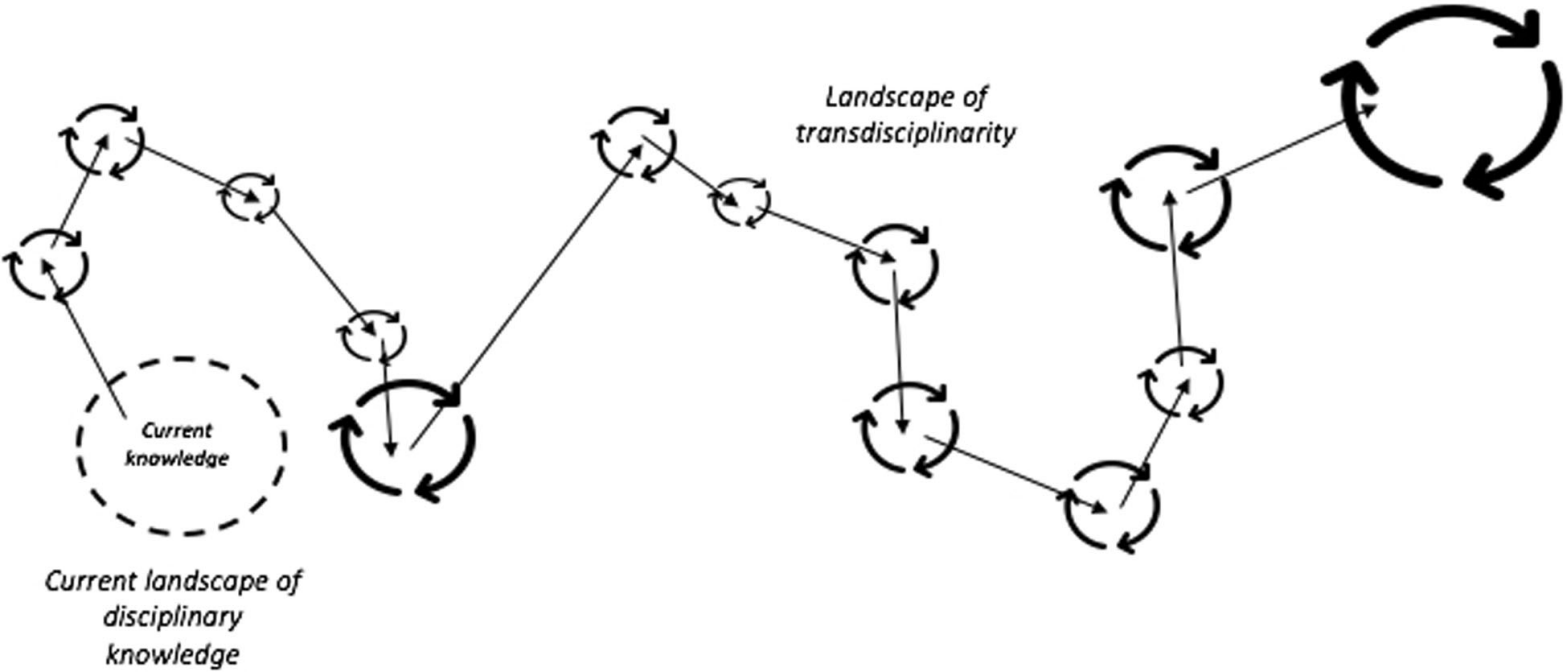
An abstraction of a hunter-gatherer model of human behaviour based on wayfinding to guide innovation. *Note:* In this model, *hunting* innovation takes individuals along an evolving path, where knowledge is grown by *gathering* information in uncharted regions experienced along the way. As there is no ‘destination’, it is this *evolving and transdisciplinary path* that ultimately instils innovation

While a schematic of a hunter-gatherer model is shown in Fig. 1, there is a need for a brief disclaimer. Notably, this behavioural model is intended to be viewed in a personal, context-dependent and dynamic way. It situates innovation as an unfolding and evolving transdisciplinary path that emerges through the pragmatic efforts made by researchers and practitioners to transition from pervasive path dependencies (such as training activities traditionally situated in ‘coach-centred’, autocratic and mechanistic pedagogies), framed by an overarching intention. Thus, this figure should be viewed as an abstraction, used to help conceptualise its principles. For example, the broken circle around the *current landscape of disciplinary knowledge* denotes its respective levels of controlled chaos and uncertainty, which would be low for the researcher and practitioner, as it is filled with regions they regularly inhabit. The traditional sub-disciplines of sport science would reside within this landscape. Comparatively, the *landscape of transdisciplinarity*, which progressively instils an innovative way of doing, is much more chaotic and uncertain, consisting of knowledge regions yet to be explored by researchers and practitioners. Note, we have intentionally not bound the uncertainty and controlled chaos of this landscape in the figure, but do wish to mention that it should be embraced by researchers and practitioners seeking transdisciplinary ways of doing, since to grow knowledge, one must wayfind through the ‘unknown’ region.

Second, the constant dynamicity of the hunt's direction reflects the anthropological ethos of *knowing as we go*. For example, the arrows represent the hunt and the circled arrows represent the gather, with the direction of the hunt being continually reorganised based on the information gathered along the way. The size of the circular arrows (gathers) reflect the arbitrary time spent inhabiting the knowledge regions—some regions are likely to afford large knowledge growth given their centrality to the innovation-prone intention (depicted by the larger circles), while others are likely to afford less knowledge growth (depicted by the smaller circles).

Lastly, while crucial to differentiate hunting and gathering behaviours, this model does not seek to categorise hunters and gatherers in sport science. Rather, *both* would be required for innovation to unfold in accord with this model [3]. It is the hunting instincts that would drive the pragmatic search and exploration through the *landscape of transdisciplinarity* framed by the innovation-prone intention (i.e., *searching for better—more effective—ways to monitor and redesign practice tasks*), while the gathering instincts would enable the discerning of how ‘new’ knowledge could be exploited to achieve the innovation-prone intention (i.e., *how can this ‘new’ knowledge be used to better redesign a practice task? How can we improve existing ways of practising, based on this knowledge source? Or, using this ‘new’ knowledge, where should we hunt next?*). The critical point here is that these hunting and gathering instincts operate symbiotically: the *hunt* informing the *gather*, and the *gather* informing the next *hunt*. As proposed by Hristovski et al. [30], this symbiosis could be understood as a type of circular causality, where future hunts are enriched by prior gathers (and vice versa), demonstrating a kind of ‘repetition without repetition’ [42].

The following sections of this paper bring life to this model, showing how it could be (figuratively) used to hunt and gather transdisciplinary knowledge to wayfind toward innovation, shaped by intentionality. To support the use of this model, three anthropological principles germane to a successful hunting and gathering party will be integrated [3]. To contextualise these principles, a threaded hypothetical example of their utility in a sports organisation is provided.

## Principle 1—*Avoid Hunting and Gathering Alone*

Anthropologically, a successful hunting-gathering party consists of a small group of individuals who share diverse knowledge, but are bound by a collective intentionality (i.e. seeking ‘prey’) [43]. This party may consist of an expert tracker, tool maker and gatherer, each integrating their knowledge during an expedition. An important consideration here is that the ‘prey’ is not likely to be stationary during the hunt, emphasising the dynamicity and requisite attunement of this behavioural model. It is the continual exploitation of environmental information detected by the party during the hunt that leads to the (re)organisation of its direction in real-time. Stated differently, the party learns *of* their ‘prey’ *as they go* by engaging with the habitat. Thus, to instantiate this behavioural model in sport, researchers and practitioners need to be mindful of the first principle common to a successful hunting-gathering party—*avoid hunting and gathering alone*.

### Principle 1 in Practice

In this example, the hunting and gathering party in a sports organisation consists of a sports coach, performance analyst, high-performance manager, and skill acquisition specialist. Through collaboration, the party have collectively identified *how to redesign practice tasks to guide and empower athlete learning and promote performance adaptability* as the overarching innovation-prone intention to shape its hunting and gathering behaviours through the *landscape of transdisciplinarity*. In this party, the sports coach and high-performance manager *gather* rich experiential and empirical knowledge to *hunt* the specific features of the game underpinning the practice tasks. The skill acquisition specialist *hunts* and *gathers* different pedagogical approaches in which the activities could be designed to guide athlete learning and promote adaptability. The performance analyst *hunts* different ways of capturing the practice activity and *gathers* this knowledge to support its ongoing redesign in real-time. Thus, while each member offers unique skills, the collaborative interaction between them shaped by the intention enables previously independent theories, ideas and/or methodologies to be enmeshed, leading to the emergence of a transdisciplinary ‘way’ of doing that is different from what they have attempted before. *The hunt has begun!*

## Principle 2—*Don’t Go Home Too Early*

Inuit hunter-gatherers would embark upon Arctic expeditions for days, weeks or months at a time, inhabiting different regions within a landscape as they found their way shaped by the collective intentionality [18]. Through this inhabitation, new information was detected and interacted with, growing knowledge, which was ultimately ‘taken home’ to enrich some aspect of practice. Importantly though, not all regions explored afforded the same richness of information to support wayfinding. Moreover, white-outs or blizzards commonly reduced the detection of information to guide the hunt, thereby prolonging the expedition [18]. The point being made here is that hunting new information and gathering translatable knowledge to wayfind through the *landscape of transdisciplinarity* will likely be a long, ongoing process, requiring persistence as the party may need to overcome emergent constraints that threaten to derail the expedition. By abandoning the journey prematurely though, the party may unwittingly miss an information-rich region just beyond their current vantage point. Thus, it is important to be mindful of the second principle germane to successful hunting and gathering—*don’t go home too early*.

### Principle 2 in Practice

Continuing from the first principle, the hunting and gathering party in the sports organisation are currently using their wayfinding skills to navigate toward new, transdisciplinary ways of designing and monitoring practice tasks to guide athlete learning and promote adaptability. In the spirit of the model, the party is following a shared ethos of *knowing as we go*, routinely reflecting upon the *gathered* knowledge following each *hunt* (i.e., attempt to redesign a practice task). It is this cyclical process of hunting and gathering that ultimately leads to the requisite transdisciplinary knowledge growth that instils innovation. To exemplify, the sports coach and high-performance manager are utilising information *gathered* by the performance analyst to continually redesign practice tasks (i.e., updating the hunt direction), while concurrently integrating the different pedagogical approaches *hunted* by the skill acquisition specialist to guide athlete learning and promote adaptability. During this process, however, the party has encountered sociocultural constraints that have threatened the innovation-prone intention. Specifically, the emergent new way of designing practice is breaking away from the traditionally engrained, disciplinary way of designing practice common to the sport and organisation, exemplified in ‘coach-centred’ practices (i.e., the coach sets the training theme, and prescribes tactical solutions to pre-determined problems designed into practice tasks). However, rather than abandoning the expedition and succumbing to these sociocultural norms, the party have persisted, actively seeking ways to overcome these constraints and continue to pragmatically refine the practice task design using their grown, transdisciplinary knowledge. This persistence is what leads them to a progressive ‘way’ of practice redesign that results in athlete learning and adaptability beyond what has been experienced by the sports organisation before. More directly, the party is evolving from a ‘coach-centred’ orientation to an ‘athlete-environment-centred’ approach—appreciating that their role is to foster athlete-environment interactions through carefully designed practice tasks that place the athlete’s needs at the core. *The ‘prey’ has been identified!*

## Principle 3—*Inhabit and Integrate*

A common strategy used by Inuit hunter-gatherers to indicate that a new region had been explored and inhabited was to create rock formations in the region that were rich in meaning [18]. These formations would indicate, for example, that a specific type of animal may reside in the area. This information would then be used to support and guide future expeditions intended to hunt that animal [18]. Importantly, to accurately design these information-rich rock formations, a hunting-gathering party would have to inhabit a region to grow their *knowledge of* it. Stated differently, to integrate a new hunting region into a landscape, a party would have to inhabit it [18]. Thus, to instil a new, innovative way of doing, researchers and practitioners in sport need to *inhabit and integrate*.

### Principle 3 in Practice

By this stage, the hunting-gathering party in the sports organisation now has a transdisciplinary way of designing and monitoring practice tasks that guides athlete learning and promotes adaptability beyond what has been done before. This new, innovative way has emerged through the *hunting* of new practice designs, and the *gathering* of knowledge to determine how well they guided athlete learning and promoted adaptability. Simply, the old way of designing practice grounded in a coach-centric pedagogy ceases to exist, with the organisation now inhabiting and integrating the new way of doing that places the athlete-environment interaction at its core. This emergent, transdisciplinary way of practice task design should not be seen as a terminus destination though. Contrarily, it is envisaged that by this stage of the expedition, the hunting and gathering party has sharpened its instincts, and the process of wayfinding through the *landscape of transdisciplinarity* has led to a new epistemology, one which has search, discover and exploit at its core, with a *knowing as we go* ethos cascading into the next innovation-prone intention. *Onto the next ‘prey’!*

## Concluding Remarks

As timelessly captured by Herman Melville’s quote at the start of this paper, the desire to explore uncharted regions in diverse landscapes is a thread that binds humans across different cultures and epochs. Using a hunter-gatherer behavioural model based on wayfinding, this paper sought to offer sport performance specialists with a conceptual basis to guide the pragmatically abductive and transdisciplinary thinking ultimately needed to instil innovation. However, it would be remiss if certain caveats of this model were not highlighted. First, it presumes that sports organisations (inclusive of academic institutions) indeed afford and prioritise ‘enough’ time for researchers and practitioners to wayfind through different knowledge regions toward the establishment of innovative ways of doing. Given a range of organisational constraints, this may not always be the case. Second, the required transdisciplinarity of this model may not be nurtured within sporting organisations that function in compartmentalised, ‘sub-disciplinary’ departments. Such organisational structures are important to progress away from when considering the use of this model, as “disciplinary fragmentation creates blind spots by framing the world in a discipline-driven way that actually prevents certain subjects from being ‘seen’” ([44], p.48). Last, the example threaded through the model should not de-limit its use to practice (re)design. Rather, this model should be seen in a much broader way, encouraging the continued prioritisation of transdisciplinarity as a mode of scientific inquiry in sport, leading toward the instilment of new and innovative ways of doing. Moreover, this model of sport science support is intended to be a guide for all researchers and practitioners seeking to discover the next idea located in a yet to be explored region—encouraging the re-discovery of hunter-gatherer instincts embedded in us all. In the spirit of transdisciplinarity, we leave our readers with a quote that eloquently captures the essence of our paper from the prominent social anthropologist, Tim Ingold ([45], p.174). His thought-provoking perspectives on knowledge and its growth continue to inspire us in sport.

“…what matters is not the final destination, but all the interesting things that occur along the way. *For wherever you are, there is somewhere further you can go*.”

## Data Availability

Not applicable

## Footnotes

1 When attempting to reach the summit of a mountain previewed from ‘sea level’, one may realise that the pre-planned route to the summit initially charted was in fact blocking the view of the actual summit further up the mountain, thereby being a *false summit*. Within innovation (and mountaineering), this could have disastrous consequences, such as the premature implementation of an idea, product or technology that leads to unseen, ill-informed and counter-productive outcomes.
